# Lifetime Prediction of PVC-P Geomembranes Immersion in Water at Elevated Temperatures

**DOI:** 10.3390/polym17111470

**Published:** 2025-05-26

**Authors:** Xianlei Zhang, Jingxin Zheng, Hesong Liu, Yunyun Wu

**Affiliations:** 1 School of Water Conservancy, North China University of Water Resources and Electric Power, Zhengzhou 450046, China; zhangxianlei@ncwu.edu.cn (X.Z.); 16634865469@163.com (J.Z.); 2 Engineering Department, Xinjiang Hami Pumped Storage Co., Ltd., Hami 839124, China; lhs6188888@163.com; 3 College of Water Conservancy and Hydropower Engineering, Hohai University, Nanjing 210024, China

**Keywords:** PVC-P geomembrane, break stress, break strain, arrhenius model, time to nominal failure

## Abstract

Plasticized polyvinyl chloride (PVC-P) geomembranes (GMBs) are applied as anti-seepage materials in membrane-faced rockfill dams and pumped storage power stations. Assessing their lifetime to ensure durability during operation is crucial. This study conducted accelerated aging tests on three PVC-P GMBs immersed in water, along with axial tensile tests to investigate the degradation of mechanical properties. The degradation model was constructed using the Arrhenius equation, and the time to nominal failure (TNF) was predicted based on this model and failure criterion. The prediction model’s accuracy was verified using test data collected over 180 days at 20 °C. The results demonstrate that the TNF of PVC-P GMBs is influenced by water temperature, plasticizer content, and thickness of GMBs. Elevated temperatures accelerate the loss rate of plasticizers. Specifically, at 20 °C in a water environment, the estimated TNFs of Materials A and B with identical thicknesses were 49.05 and 153.76 years, respectively. This suggests that increasing the initial plasticizer content and enhancing its structural stability can significantly extend the TNF. Furthermore, Material C, which has a composition similar to Material B but with increased thickness, exhibited a predicted TNF of 181.30 years, indicating that greater thickness can effectively reduce the migration rate of plasticizers. The findings provide a theoretical basis for evaluating the TNF of PVC-P GMBs in reservoir bottom and below dead water level applications during operation.

## 1. Introduction

Plasticized polyvinyl chloride (PVC-P) geomembranes (GMBs) are characterized by their outstanding flexibility, exceptional impermeability, ease of construction, and cost-effectiveness. Consequently, they have been widely applied in various projects, including dams, reservoir basins, landfills, and tunnels [1,2,3,4,5,6]. According to Bulletin 135 by the International Commission on Large Dams (ICOLD), PVC-P GMBs account for 76% of GMBs as principal anti-seepage materials in 176 membrane-faced rockfill dams [7]. The development of pumped storage power stations in China has highlighted the limited deformation adaptability, challenges in underwater repair, and high costs associated with relatively rigid concrete or asphalt concrete slabs of large area [8,9]. Although polyethylene (PE) or high-density polyethylene (HDPE) GMBs exhibit ultra-low permeability and durability, their adaptability to local deformation diminishes as thickness increases [10]. Consequently, PVC-P GMBs with superior flexibility are preferred as anti-seepage materials within reservoir basins. Given that pumped storage power stations are designed for a lifespan of approximately 100–150 years, it is imperative that PVC-P GMBs remain effective throughout this period. Field and laboratory data suggest that the mechanical properties of PVC-P GMBs degrade over time due to aging. Thus, assessing the long-term performance and deducing the time to nominal failure (TNF) of these GMBs under typical exposure conditions is crucial.

There are two ways for assessing the TNF of GMBs mentioned in the literature, either in situ samplings or laboratory testing. What is at the heart of this paper is laboratory-accelerated aging tests. Prior studies have demonstrated that the severity of GMBs aging is influenced by exposure media (e.g., air, water, leachate, hydrocarbons, acid mine drainage) and temperature [11]. Koerner et al. [12] incubated HDPE GMBs at 80, 70, and 60 °C until a 50% reduction of strength and elongation occurred, subsequently calculating the half-life value at 20 °C. Lodi et al. [13] examined the effects of weathering exposure on mechanical properties, tear, and puncture resistance of HDPE and PVC GMBs. Müller and Jacob [14] studied the degradation behavior of HDPE GMBs by exposing them to a forced air oven and immersion in a flask placed in a temperature-controlled oven at 80 °C. They found that antioxidants depleted significantly faster in water than in air. Ewais et al. [15] investigated the degradation of HDPE GMBs aged at 55, 70, and 85 °C in air, water, and leachate, predicting their lifetime using the Arrhenius equation. Rowe et al. [16] conducted an accelerated aging study on HDPE GMBs immersed in air, distilled water, and synthetic leachate at temperatures ranging from 85 °C to 22 °C (room temperature) and concluded that antioxidant depletion rates were fastest for samples exposed to leachate, followed by water with the slowest depletion occurring in air. Abdelaal et al. [17,18] investigated the long-term performance of HDPE GMBs stabilized with HALS and without HALS by immersing the GMB coupons in chlorinated water at temperatures up to 85 °C. Arrhenius modeling was used to predict the antioxidant depletion time, and the TNF and severe degradation based on SCR. Clinton and Rowe [19] examined the changes in the physical-mechanical properties of HDPE GMBs immersed in leachate at five temperatures (40, 65, 75, 85, 95 °C), utilizing Arrhenius modelling to predict the TNF. Samea and Abdelaal [20] examined the degradation behavior of elastomeric bituminous GMBs exposed to air and deionized water at temperatures between 22 and 85 °C, finding that exposure to deionized water resulted in faster degradation of mechanical properties.

Numerous methods for predicting the TNF of polymers have been established. Literature [14,15,16,17,18,19,20] employed Arrhenius modelling to estimate the TNF. Plota and Masek [21] described various methods for lifetime prediction, including the Arrhenius model, time-temperature superposition, and Williams–Landel–Ferry model. Cazzuffi and Gioffrè [22] conducted a 25-year ongoing study on the long-term performance of PVC-P GMBs installed on the upstream face of Italian dams, and predicted the lifetime of exposed GMBs using the function of the loss of plasticizers. Luciani et al. [6] designed an accelerated aging test device to perform mechanical and absorption tests on two PVC-P GMBs at 45, 60 and 75 °C, and derived a plasticizer loss rate of 0.45 at the threshold of effective performance. Blanco et al. [23] evaluated the durability of PVC-P GMBs installed in irrigation reservoirs for 18–31 years utilizing the loss rate of plasticizers and found that the loss rate of plasticizers ranged from 71.0% to 84.3%, greater than the threshold referred to in literature (45%, 30%). However, the commonly used lifetime prediction model for GMBs is the Arrhenius model [24,25,26,27].

In summary, extensive research has been conducted on the durability of GMBs, with a primary focus on HDPE GMBs. Literature regarding the long-term performance of PVC-P GMBs has primarily examined thermal aging and ultraviolet aging. Studies like this are extremely important for geosynthetics serving critical functions like waterproofing of dams. However, such lifetime prediction is only for the particular formulation that has been evaluated. Transferability of those results to other formulations is neither realistic nor appropriate [11]. Previous studies have demonstrated that the main degradation mechanism of PVC-P GMBs is plasticizer loss. For underwater applications in reservoirs, while the aqueous environment does not dissolve the organic plasticizers, it may accelerate their migration or loss. Therefore, assessing the durability and TNF of PVC-P GMBs immersed in water is of significant importance.

The primary objective of this paper is to predict the TNF and assess the durability of three PVC-P GMBs for underwater applications in reservoirs. The objectives are divided into the following parts: (1) Carry out laboratory accelerated aging tests combined with axial tensile tests to investigate the degradation of mechanical properties of PVC-P GMBs. (2) Establish the failure criterion and construct degradation models. (3) Predict the TNF of PVC-P GMBs at operating temperatures based on the developed degradation models. (4) Validate the model using the CORREL statistical function.

## 2. Materials and Methods

### 2.1. Materials

Three commercially available PVC-P GMBs, manufactured by different domestic producers, were selected for this study. Each roll measured 45.0 m in length and 2.0 m in width. These materials are hereafter referred to as Material A, Material B, and Material C, respectively. The properties of PVC-P GMBs are reported in Table 1.

The PVC-P GMB is primarily composed of PVC resin, plasticizer, filler, heat stabilizer, ultraviolet absorbent, and antioxidant. Material B and Material C have the same molecular structure, composition, and mass proportion but differ in thickness. The composition and mass proportion of the three PVC-P GMBs are summarized in Table 2.

### 2.2. Test Equipment

The test equipment mainly consists of an electric thermostatic water tank, a tensile testing machine for geosynthetics, and a thermogravimetric analyzer.

(1) The apparatus for water immersion tests is an electric thermostatic water tank (HH600), produced by Soper Instruments Co., Ltd (Shaoxing, China). The temperature ranges from room temperature to 99 °C with an accuracy of 0.2 °C.

(2) The tensile testing machine for geosynthetics (CMT5000, Nss (Shenzhen) Laboratory Equipment Co., Ltd., Shenzhen, China) was employed to perform the axial tensile tests, with parameters as follows: a maximum load of 30.0 kN, a maximum stroke of 2.1 m, and displacement ranging from 0.2% to 100% of the maximum stroke with an error in 0.5%.

(3) The thermogravimetric analyzer, model TG 209 F1 Libra (NETZSCH-Gerätebau GmbH, Schelbe, Germany), with a temperature range of 10 to 1100 °C, a temperature increase rate of 0.001 to 200 K/min, a maximum weighing range of 2000 mg, and a resolution of 0.1 µg.

### 2.3. Test Methods

**Figure 1:**
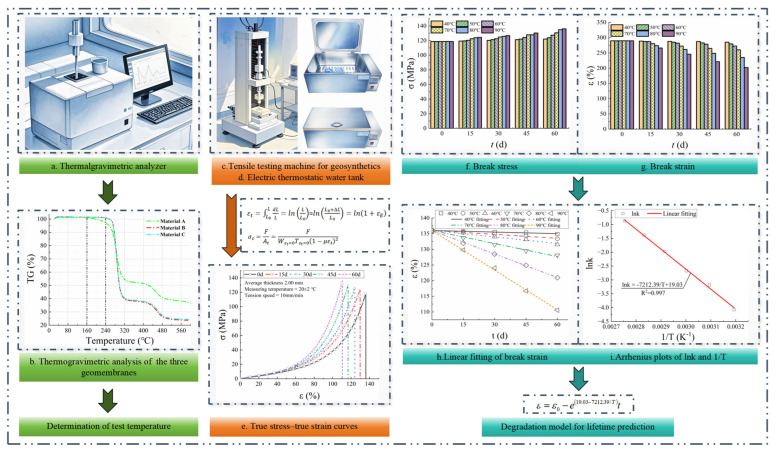
Procedure for establishing the degradation model for lifetime prediction of PVC-P GMBs.

The procedure for establishing the degradation model for lifetime prediction of PVC-P GMBs is illustrated in Figure 1. It primarily consists of three steps: (1) The thermogravimetric analysis was performed using a thermogravimetric analyzer (Figure 1a) to determine the appropriate test temperatures. (2) The GMB coupons were immersed in thermostatic water tanks maintained at the specified test temperatures. Axial tensile tests were conducted on specimens retrieved after varying incubation durations to acquire true stress-strain curves. (3) The break stress and strain data were analyzed to develop a degradation model based on the Arrhenius equation.

#### 2.3.1. Determination of Test Temperatures

**Figure 2:**
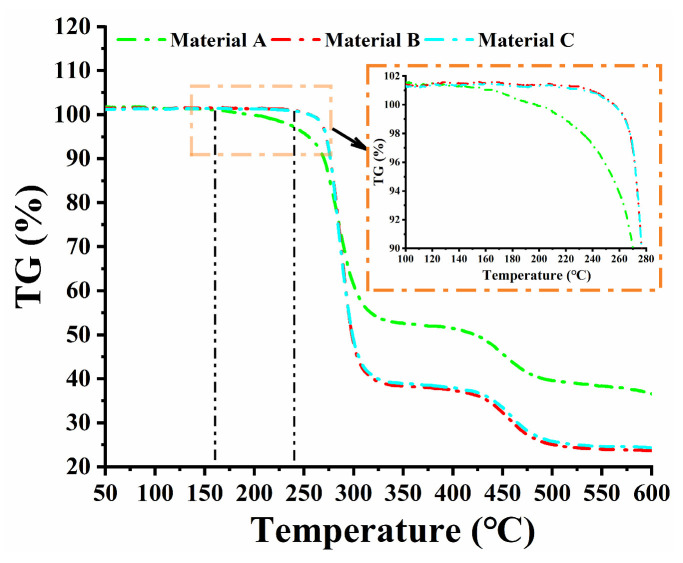
Thermogravimetric analysis of GMBs.

The selected temperatures should be: (1) high enough to accelerate the aging process and practically allow aging evaluation in a reasonable period, (2) not so high to modify the molecular structure of the material. The temperatures were selected based on thermogravimetric analysis performed on three PVC-P GMBs. Figure 2 depicts the thermogravimetric curves of three materials. The weight percentage of Material A decreased slowly in the range of 0 to 162 °C, while that of Materials B and C remained stable in the range of 0 to 240 °C, indicating a small amount of plasticizer loss in the GMBs. The PVC resin and other components began to decompose at temperatures higher than 162 °C and 240 °C, resulting in a sharp decline in weight percentage and then failure of the molecular structure of the GMBs. Consequently, to avoid the breakage of the chemical bonds of PVC, combined with the feasibility of the water immersion tests, accelerating aging temperatures were set at 20, 40, 50, 60, 70, 80, and 90 °C, consistent with the ones mentioned in previous literature on this topic [15,16,17,18,19,20].

#### 2.3.2. Accelerating Aging Tests

The primary contents of accelerating aging tests are detailed as follows:

(1) The immersion tests were conducted in thermostatic water tanks at temperatures of 20, 40, 50, 60, 70, 80, and 90 °C, and set the duration of 60 days except for 180 days at 20 °C (The experimental data at 20 °C were used to validate the degradation models). Four GMB coupons with dimensions of 20 × 20 cm were immersed at each temperature condition to ensure sufficient specimens for tensile testing, and they were separated by 5 mm diameter glass rods to ensure exposure of GMB to water from both sides. At an interval of 15 days, a GMB coupon was retrieved, dried at 85 °C, and then maintained at room temperature for 24 h before tensile tests. The water was replaced every 5 days, and the water tanks and the GMBs were cleaned to prevent the build-up of migrated plasticizers in the water.

(2) After immersion, the GMB coupon was cut into five dumbbell-shaped specimens (Type Ⅳ) for each temperature. The PVC-P specimens were examined at 20 ± 2 °C in accordance with ASTM D6693/D6693M-20 [30] at a tensile rate of 10 mm/min to monitor the degradation of mechanical properties with time.

## 3. Results

### 3.1. Analysis Methods

The stress is calculated as follows:

(1) $$\sigma_{E}=\frac{F}{A}$$

where $\sigma_{E}$ is the stress, F is the measured force acting on specimen during the uniaxial tensile test, and A is the cross-sectional area of specimen.

The strain is calculated as follows:

(2) $$\varepsilon_{E}=\frac{∆L}{L_{0}}=\frac{L-L_{0}}{L_{0}}$$

where $\varepsilon_{E}$ is the strain, ∆L is the increment of gauge length during the uniaxial tensile test, $L_{0}$ is the initial gauge length of specimen, and L is the distance between gauge marks at any time.

Equation (1) was based on the assumption that the cross-sectional area remains constant during the tensile test. However, the cross-sectional area of the specimen changes during the tensile process. Consequently, the stress-strain calculated cannot accurately reflect the mechanical characteristics of the GMBs. Therefore, the true stress and strain equations were derived by Merry and Bray [31].

The true strain is expressed as follows:

(3) $$\varepsilon_{t}=\int_{L_{0}}^{L}\frac{dL}{L}=\ln\left(\frac{L}{L_{0}}\right)=\ln\left(\frac{L_{0}+\Delta L}{L_{0}}\right)=\ln\left(1+\varepsilon_{E}\right)$$

where $\varepsilon_{t}$ is the true strain.

The true stress is expressed as follows:

(4) $$\sigma_{t}=\frac{F}{A_{t}}=\frac{F}{W_{\varepsilon_{t}=0}T_{\varepsilon_{t}=0}{\left(1-\mu \varepsilon_{t}\right)}^{2}}$$

where $\sigma_{t}$ is the true stress, $A_{t}$ is the cross-sectional area of specimen during the uniaxial tensile test, $W_{\varepsilon_{t}=0}$ is the initial width of specimen, and $T_{\varepsilon_{t}=0}$ is the initial thickness of specimen, μ is the Poisson’s ratio (0.5).

### 3.2. True Stress-True Strain Curves

**Figure 3:**
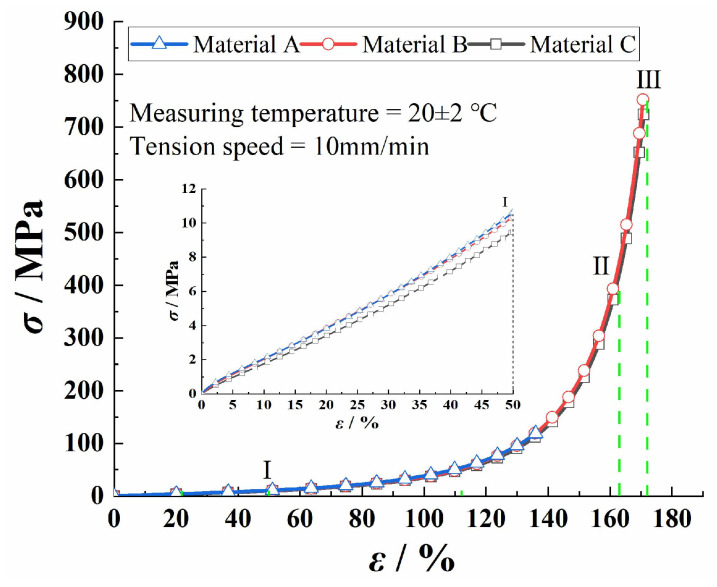
True stress-true strain curves for the three PVC-P GMBs.

Figure 3 shows the true stress-true strain curves of the three PVC-P GMBs. These curves can be typically classified into three distinct stages. In the initial stage (0–I), a linear relationship is observed between the stress and strain. During the second stage (I to II), referred to as the yield stage, the stress grows rapidly with strain, exhibiting a nonlinear and dramatically bending curve. In the final stage II to III), known as the strengthening stage, the stress-strain relationship becomes virtually linear again, with a significantly increased slope, until the specimen ultimately fractures.

**Figure 4:**
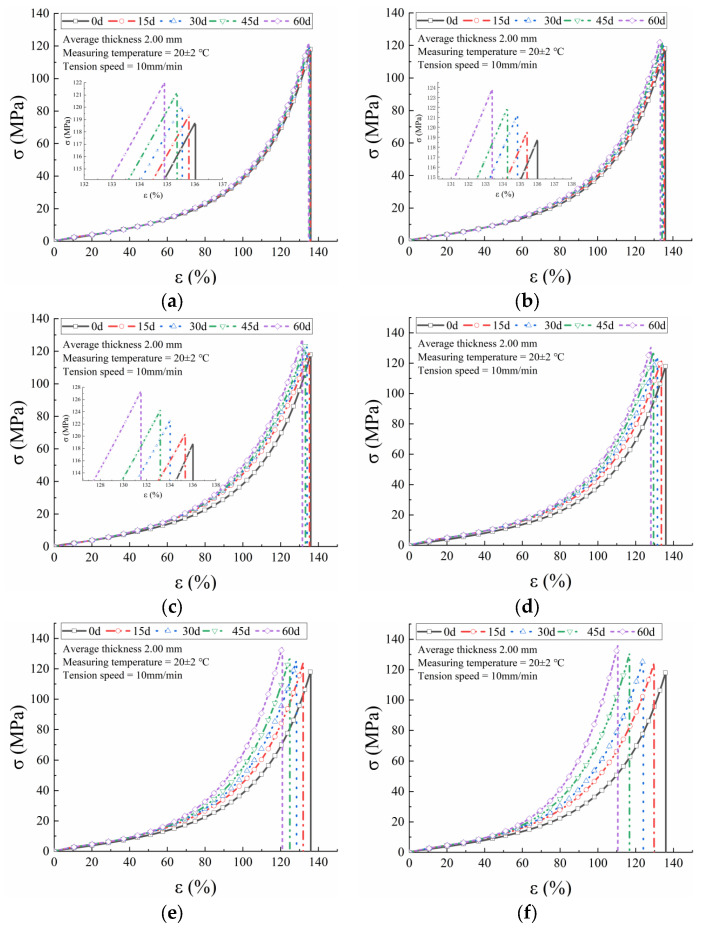
True stress–strain curves for Material A at different temperatures. (**a**) 40 °C; (**b**) 50 °C; (**c**) 60 °C; (**d**) 70 °C; (**e**) 80 °C; (**f**) 90 °C.

Because the true stress-strain curves for the three GMBs exhibit similar trends, only the curves for Material A at temperatures ranging from 40 to 90 °C are presented in Figure 4. As evident, the slope of the curve during the initial stage increased with temperature across all tested temperatures. Additionally, the break strain decreased while the break stress increased. For Materials B and C, the mechanical properties exhibited no significant difference as the temperature increased, indicating their superior thermal stability. The true stress-strain curves at the same temperature but different durations remained very close, particularly, coinciding almost perfectly at 40 °C. All three PVC-P GMBs exhibited closely aligned curves over the duration of 0–60 days at 40 °C, with the greatest departures observed at 90 °C.

### 3.3. Yield Strain and Yield Stress

**Figure 5:**
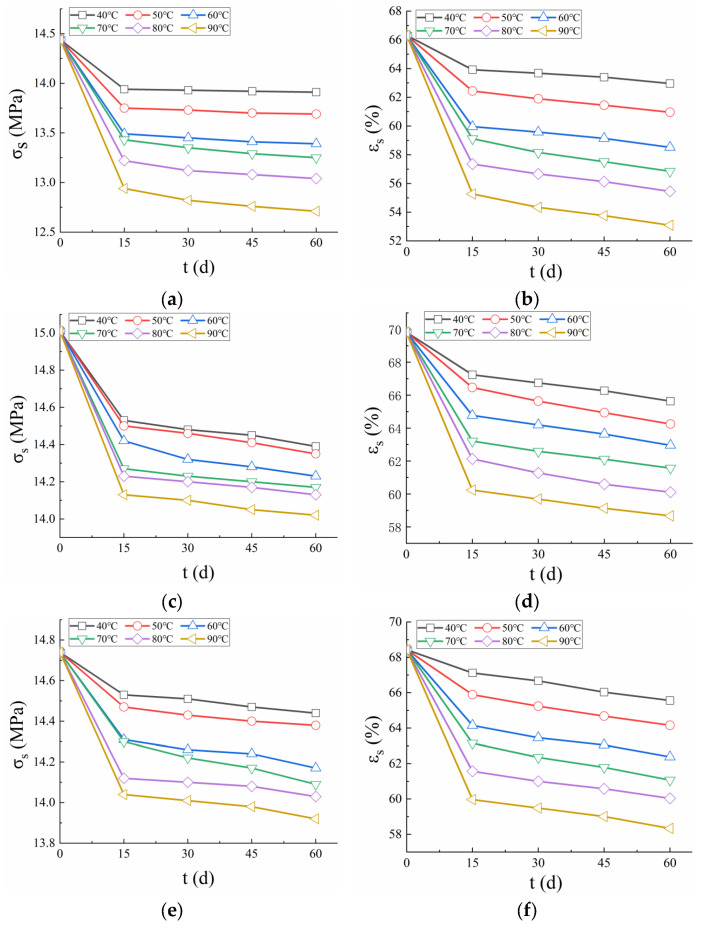
Yield stress and strain of the three PVC-P GMBs. (**a**) yield stress of Material A; (**b**) yield strain of Material A; (**c**) yield stress of Material B; (**d**) yield strain of Material B; (**e**) yield stress of Material C; (**f**) yield strain of Material C.

Figure 5 illustrates the variation in yield strain and stress over time at different temperatures for the three PVC-P GMBs. The results demonstrated that both yield strain and yield stress exhibited a decreasing trend as test temperature and duration increased, indicating a degradation of mechanical properties under high-temperature water exposure. After being immersed in water at 40 °C for 15 days, the yield stress of Material A decreased from 14.44 to 13.94 MPa (a reduction of 3.46%), and the yield strain decreased from 66.31% to 63.92% (a reduction of 3.6%). By 60 days, the yield stress further decreased to 13.91 MPa, and the yield strain to 62.96%, with both parameters showing a reduction of less than 2%. At 90 °C, within the same 15-day period, the yield stress and yield strain of Material A decreased by 10.39% and 16.65%, respectively, which were significantly higher reductions compared to those observed at 40 °C. This indicates that elevated temperatures accelerate the degradation of mechanical properties. For Material B, at 40 °C, the yield stress and yield strain decreased by 3.2% and 3.71% over the initial 15 days, and by 0.96% and 2.38% over the subsequent 45 days (up to 60 days). For Material C, the corresponding decreases were 1.4% and 1.9% over the initial 15 days, and 0.62% and 2.32% over the next 45 days. Within the initial 15 days, there was a significant reduction in yield strain and stress, followed by a period of slower decline. This behavior suggests that the PVC-P GMBs were initially highly sensitive to the water environment, but their rate of performance degradation diminished as they gradually adapted to the conditions over time.

Furthermore, the findings revealed that Materials B and C generally maintained higher yield strain and stress compared to Material A. This suggests that Materials B and C experienced less plasticizer loss during testing, resulting in superior ductility and making them more suitable for application in complex aquatic environments.

### 3.4. Break Stress and Strain

**Figure 6:**
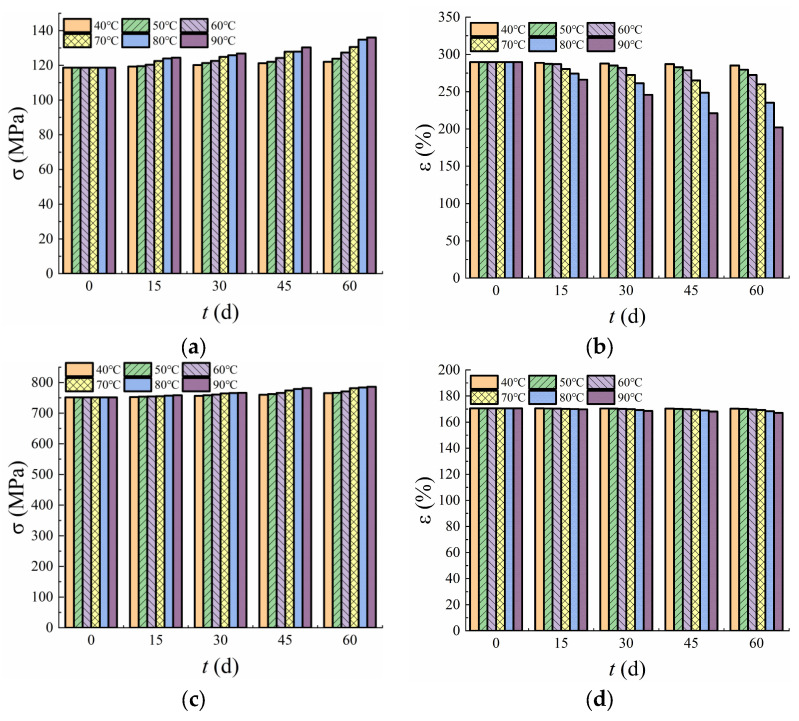

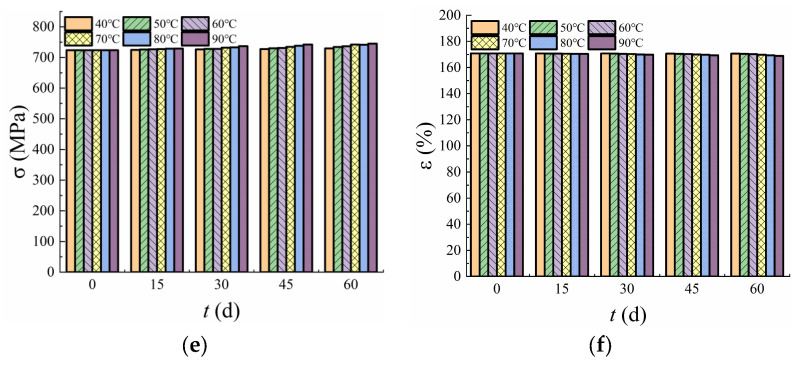
Break stress and strain of the three PVC-P GMBs. (**a**) break stress of Material A; (**b**) break strain of Material A; (**c**) break stress of Material B; (**d**) break strain of Material B; (**e**) break stress of Material C; (**f**) break strain of Material C.

Figure 6 illustrates the variation in break stress and strain over time at test temperatures. The break stress of all three materials increased with both time and temperature, with the rate of increase becoming more pronounced as the temperature rose. At 40 °C, the break stress of Material A increased from 118.69 MPa to 122.01 MPa over a period of 60 days, representing a 2.8% increase. At 90 °C, the break stress increased from 118.69 MPa to 136.04 MPa, corresponding to a significant 14.62% increase. This comparison indicates that temperature elevation has a more pronounced effect on break stress than the duration of exposure. At 40 °C, the break stress of Materials B and C increased by 1.8% and 0.8%, respectively. The greater increase in break stress observed for Material A compared to Materials B and C suggests that Material A exhibits inferior stability relative to the other two GMBs.

The break strain for all materials decreased over time at all test temperatures, with the decrease rate positively correlated with the test temperature. At 40 °C, the break strain of Material A declined from 289.71 to 285.37 over an immersion time of 60 days, decreasing by 1.5%. At 90 °C, the break strain decreased to 202.14, decreasing by 30.23%. Similarly, at 40 °C, the break strain of Materials B and C decreased by 0.18% and 0.12%, respectively. Material A showed a notably higher degradation rate of break strain compared to Materials B and C. Among these, Material B had a slightly higher degradation rate than Material C, suggesting that Materials B and C have superior aging resistance compared to Material A. Furthermore, the aging resistance of Material C with a thickness of 2.5 mm was better than that of Material B with a thickness of 2.0 mm, indicating that increasing the thickness of PVC-P GMBs can reduce the aging rate and extend their TNFs.

In conclusion, the mechanical properties of Material A followed similar patterns to those of Materials B and C in response to varying test durations and temperatures, characterized by a continuous increase in break stress and a continuous decrease in break strain within the temperature range of 40–90 °C. Additionally, an increase in test temperature accelerated both the growth rate of break stress and the degradation rate of break strain.

## 4. Discussion

### 4.1. Failure Criterion

The nominal failure of GMBs is typically defined as a point at which a critical mechanical property degrades to an arbitrary threshold, often assumed to be 50% of the initial value or 50% of the specified value [15,16,25,32]. Hsuan et al. [11] proposed the tensile break elongation and strength to predict the lifetime of PVC-P GMBs. Koerner et al. [12] predicted the TNF of GMBs using a 50% reduction in tensile strength and elongation. Silva et al. [33] predicted the TNF of the HDPE GMB based on break strength or elongation. Blanco et al. [34] evaluated EPDM GMBs using the elongation at break as the primary criterion. In an impervious structure, the PVC-P GMB should not only fulfill impermeability requirements but also accommodate uneven foundation settlement. Given that break strain exhibits greater sensitivity to degradation compared to break stress and is more indicative of long-term performance, it provides more conservative estimates of the TNF. Therefore, this study adopts a 50% reduction in break strain as the failure criterion for prediction of TNF.

### 4.2. Degradation Model

Zero-order, first-order, and second-order degradation kinetic equations were employed to fit the experimental data of break strain. The fitting results using a zero-order kinetic equation exhibited the highest coefficient of determination (R^2^). Consequently, the results of break strain for immersion in water were fitted using two-parameter (zero order) degradation kinetic equations, namely:

(5) $$\varepsilon \left(t\right)=\varepsilon_{0}-kt\;$$

where $\varepsilon \left(t\right)$ is the break strain as a function of time, $\varepsilon_{0}$ is the break strain of virgin PVC-P GMB, k is the degradation rate of PVC-P GMB, and t is the aging time.

**Figure 7:**
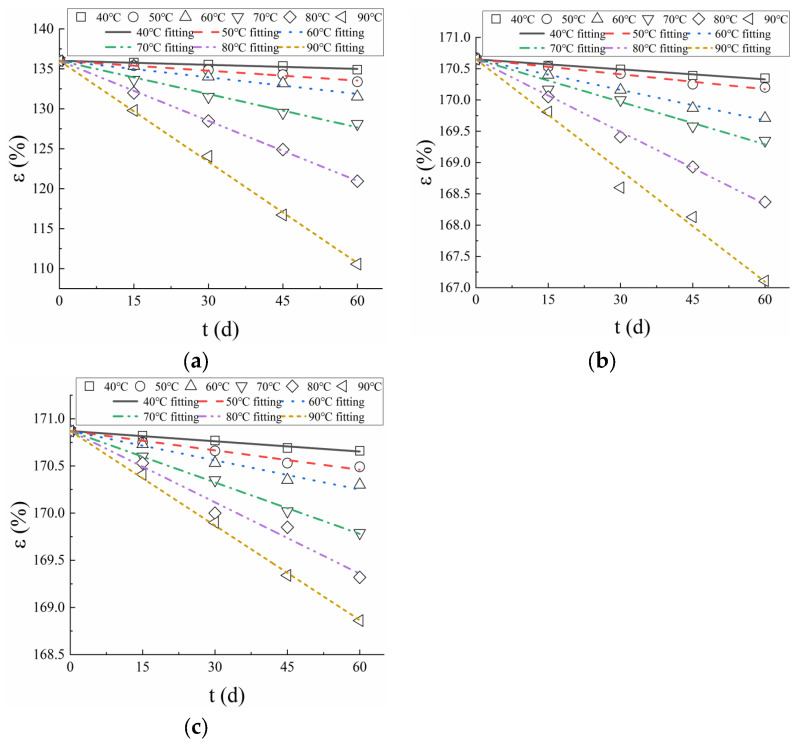
Linear fitting of break strain: (**a**) Material A; (**b**) Material B; (**c**) Material C.

A linear regression of the data (Equation (5)) yields slopes that are the degradation rates for each temperature as summarized in Table 3. Figure 7 presents the fitting results of the break strain of PVC-P GMBs. The coefficient of determination R^2^ of the regression line ranged from 0.959 to 0.995, indicating that Equation (5) fits well with the experimental data. It is evident that the degradation rates are higher at high temperatures than at low temperatures.

The degradation rates obtained at different temperatures were used to establish predictions of the degradation of the GMBs at different field temperatures. The Arrhenius equation is one of the best-known models for assessing the lifetime of polymers, as it allows short-term tests conducted at elevated temperatures to be used to assess long-term exposures at lower temperatures. The temperature dependence of the degradation rate can be described by Arrhenius equation:

(6) $$k=Ae^{-\frac{E_{a}}{RT}}$$

where A is a constant, $E_{a}$ is the activation energy, T is the absolute temperature, and R is the universal gas constant.

Taking the natural logarithm of both sides of Equation (6) yields:

(7) $$\ln k=\ln A-\frac{E_{a}}{R}\cdot \frac{1}{T}$$

**Figure 8:**
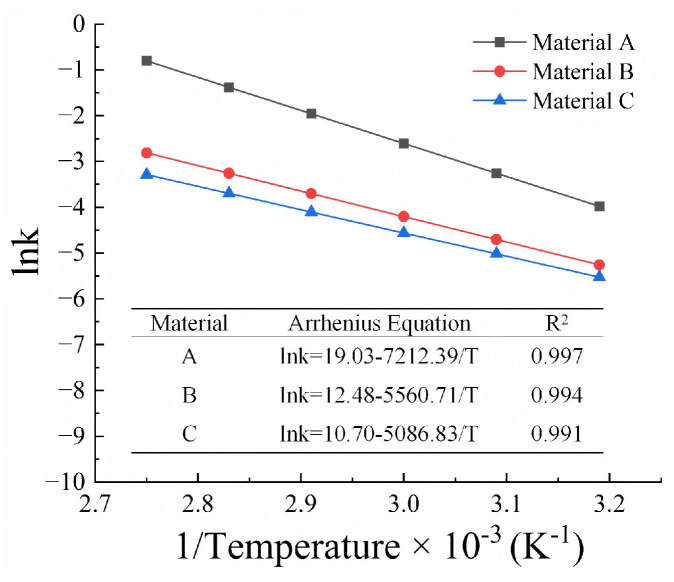
Arrhenius plots of the break strain.

The parameters in the Arrhenius equation (Equation (7)) can be established by obtaining a best fit to the data plotted in terms of the logarithm of degradation rate versus the inverse of the temperature using linear regression. Figure 8 shows the Arrhenius plots of the break strain of PVC-P GMBs. The correlation coefficients were greater than 0.99 for all the examined PVC-P GMBs, demonstrating that the Arrhenius equation is applicable to the lifetime prediction of PVC-P GMBs.

Based on the fitted parameters and Equations (5) and (6), the degradation models of PVC-P GMBs were determined as follows:

Material A:

(8) $$\varepsilon =\varepsilon_{0}-e^{\left(19.03-7212.39/T\right)}\;t$$

Material B:

(9) $$\varepsilon =\varepsilon_{0}-e^{\left(12.48-5560.71/T\right)}\;t$$

Material C:

(10) $$\varepsilon =\varepsilon_{0}-e^{\left(10.70-5086.83/T\right)}\;t$$

### 4.3. Prediction of TNF

An average temperature of 20 °C is adopted as the field temperature for prediction of TNF based on Arrhenius model. Based on the degradation model and the failure criterion, the predicted TNFs of Materials A, B, and C at 20 °C were 49.05, 153.76, and 181.30 years, respectively. The TNFs of Materials B and C were 3.13 and 3.70 times longer than that of Material A, respectively. For materials with the same thickness of 2.0 mm, Material A, containing a lower plasticizer content, exhibited a shorter TNF compared to Material B. With the virtually identical plasticizer content, Material C with a greater thickness had a slightly longer TNF than Material B. As aging initiated from the surface and progressed inward, a greater thickness in Material C resulted in a reduced degradation rate.

Therefore, working temperature served as an external factor influencing TNF, while plasticizer content and stability were fundamental determinants of longevity.

### 4.4. Model Validation

To validate the lifetime prediction model, the PVC-P GMBs were tested at 20 °C for varying durations of 10, 15, 30, 45, 60, 100, 105, 120, 135, 150, 165, and 180 d. Then the break strains of the three PVC-P GMBs were predicted using Equations (8)–(10), respectively. The CORREL statistical function was employed to analyze the correlation between the measured and predicted break strains, thereby evaluating the accuracy of the prediction model. The correlation coefficient (r) reflects the degree of linear association between the two sets of data; r = 1 indicates a perfect positive linear correlation, r = −1 indicates a positive negative correlation, and r = 0 indicates no correlation.

**Figure 9:**
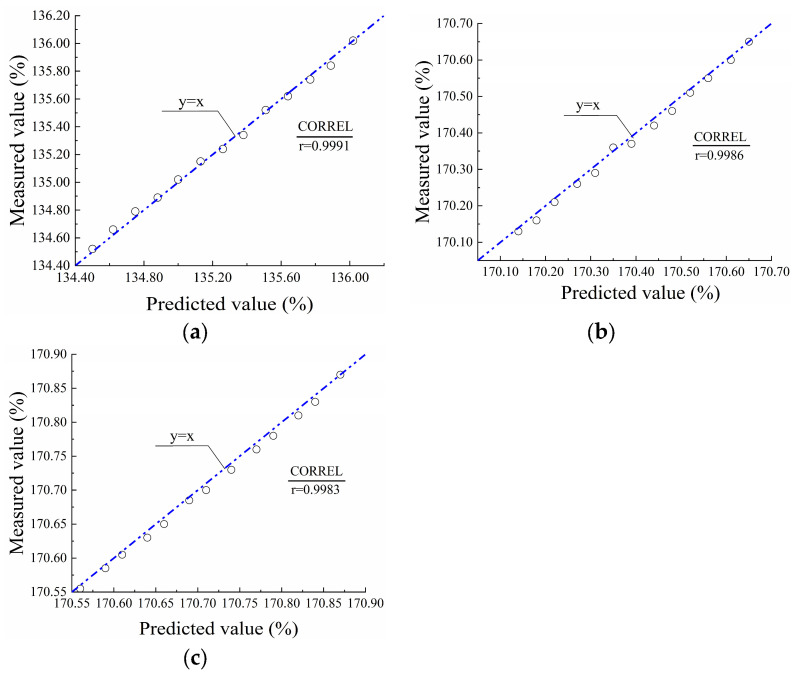
Comparison between the measured and predicted break strains: (**a**) Material A; (**b**) Material B; (**c**) Material C.

Figure 9 presents a comparison between the measured and predicted break strains for the three PVC-P GMBs at 20 °C. As can be observed, the predicted values exhibited excellent agreement with measured values. The coefficients of determination ranged from 0.9983 to 0.9991, indicating a strong linear correlation between the predicted and measured values. This high level of correlation verifies the reliability of the lifetime prediction model for the PVC-P GMBs immersed in water.

## 5. Conclusions

This paper presents an investigation into the durability of three PVC-P GMBs through accelerated aging tests. In accordance with the experimental results, the mechanical properties were analyzed, the Arrhenius model was constructed, and the TNF was predicted. For the GMBs and conditions examined, the following conclusions can be reached:

(1) PVC-P GMBs exhibited degradation in mechanical properties when immersed in water at elevated temperatures. Their adaptability to deformation exhibited a decreasing trend, with the mechanical indices such as break strain, yield strain, and yield strength continuously declining with both time and temperature. Conversely, break stress exhibited an increasing trend.
(2) The degradation of break strain over time complies with the zero-order degradation kinetic reaction and can be characterized by Arrhenius equation. The failure criterion and degradation model can accurately predict the TNF of PVC-P GMBs immersed in water at field temperatures, providing a theoretical basis for evaluating the TNF of PVC-P GMBs in water environments for a long time.
(3) According to the degradation model, the TNFs of Materials A, B, and C at 20 °C were 49.05, 153.76, and 181.30 years, respectively. This indicates that increasing the internal plasticizer content and thickness of the GMBs can reduce plasticizer loss and can prolong their TNF. However, improving plasticizer stability is the fundamental solution to extending the TNF.
(4) A comparison between the measured and predicted break strains demonstrated a high level of correlation, verifying the reliability of the lifetime prediction model for PVC-P GMBs immersed in water.

This study allows an assessment of how elevated temperatures affect the degradation of PVC-P GMBs. The findings provide considerable insight into the degradation behaviour of PVC-P GMBs. Although the prediction of TNF was satisfactory in this study, certain deficiencies remain. For example, the axial tensile test cannot accurately reflect the actual engineering practice. Bi-directional or multiple tensile tests, pullout tests, interface shearing tests, creep rupture tests, cyclic fatigue tests, and direct shear tests should be conducted to investigate the mechanical properties of PVC-P GMBs immersed in water in the future. Additionally, the plasticizer loss was not quantified. Accordingly, chromatography or mass spectrometry analyses could be performed to reveal the relationship between plasticizer loss and evaluation indicators for lifetime predictions. Furthermore, the predictions presented for PVC-P GMBs immersed in water were established based on immersion tests involving double-sided exposure of the GMB to water that are more aggressive than field exposure conditions in which the GMB is merely exposed to water from the top surface. Thus, the predicted TNFs herein are expected to be more conservative (i.e., shorter) than those in the field.

## Figures and Tables

**Table 1:** Properties of tested PVC-P GMBs.

Property	Method	Value			Unit
		Material A	Material B	Material C	
Thickness	ASTM D5199-12 [28]	2.0	2.0	2.5	mm
Mass per unit area	ASTM D5261-10 [29]	0.281	0.261	0.366	g/cm^2^
Break strength	ASTM D6693/D6693M-20 [30]	118.69	751.74	723.81	MPa
Yield strength	ASTM D6693/D6693M-20 [30]	14.44	15.01	14.74	MPa
Break elongation	ASTM D6693/D6693M-20 [30]	136.02	170.65	170.87	%
Yield elongation	ASTM D6693/D6693M-20 [30]	66.31	69.83	68.43	%

**Table 2:** Composition and mass proportion of PVC-P GMBs.

Composition and Mass Proportion (%)	Material A	Material B	Material C
PVC resin	57.21	54.88	55.11
Plasticizer (DOP ①)	20.32	32.63	32.71
Fillers	19.32	8.32	8.24
Heat stabilizer ②	1.31	2.14	2.14
Antioxidant	1.21	1.32	1.30
Ultraviolet absorbents	0.51	0.71	0.50
Other additives	0.12	-	-

NOTES: ① DOP refers to dioctyl phthalate, ② heat stabilizer is mainly calcium stabilizer.

**Table 3:** Linear fitting results for the break strain.

Temperature/°C	Material A		Material B		Material C	
	k/d^−1^	R^2^	k/d^−1^	R^2^	k/d^−1^	R^2^
40	0.017	0.991	0.053	0.995	0.036	0.990
50	0.041	0.994	0.080	0.992	0.068	0.987
60	0.069	0.993	0.163	0.990	0.104	0.976
70	0.139	0.990	0.226	0.985	0.182	0.991
80	0.251	0.995	0.386	0.976	0.252	0.959
90	0.422	0.991	0.592	0.965	0.334	0.989

## Data Availability

The original contributions presented in this study are included in the article. Further inquiries can be directed toward the corresponding authors.

## References

[1] Girard H., Fischer S., Alonso E. (1990). Problems of friction posed by the use of geomembranes on dam slopes-Examples and measurements. Geotext. Geomembr..

[2] Whitfield B. (1996). Geomembrane application for an RCC dam. Geotext. Geomembr..

[3] Touze N. (2021). Healing the world: A geosynthetics solution. Geosynth. Int..

[4] Newman E.J., Stark T.D. (2009). Ten-year PVC geomembrane durability. Geosynth. Int..

[5] Rowe R.K. (2011). Systems engineering: The design and operation of municipal solid waste landfills to minimize contamination of groundwater. Geosynth. Int..

[6] Luciani A., Todaro C., Martinelli D., Peila D. (2020). Long-term durability assessment of PVC-P waterproofing geomembranes through laboratory tests. Tunn. Undergr. Space Technol..

[7] ICOLD (2010). Geomembrane Sealing Systems for Dams-Design Principles and Review of Experience.

[8] Deng G., Wang X., Wen Y., Yu S., Chen R. (2015). Study on conceptualization method of deformation pattern and horizontal breakage of face slab of concrete faced rockfill dam. J. Hydraul. Eng..

[9] Wang Y., Wang D., Zhao L. (2021). Study on the aging of asphalt concrete facings sealing layer and the treatment countermeasures during reservoir operation period. Water Power..

[10] Zhang X.L., Ma Z.Y., Wu Y.Y. (2023). Mechanical properties of different geomembranes in membrane-faced rockfill dam. Chin. J. Geotech. Eng..

[11] Hsuan Y.G., Schroeder H.F., Rowe R.K., Müller W., Greenwood J., Cazzuffi D., Koerner R.M. Long-term performance and lifetime prediction of geosynthetics. Proceedings of the EuroGeo 4-4th European Geosynthetics Conference.

[12] Koerner R.M., Hsuan Y.G., Koerner G.R. (2017). Lifetime predictions of exposed geotextiles and geomembranes. Geosynth. Int..

[13] Lodi P.C., Bueno B.S., Vilar O.M. (2013). The effects of weathering exposure on the physical, mechanical, and thermal properties of high-density polyethylene and poly (vinyl chloride). Mater. Res..

[14] Müller W., Jakob I. (2003). Oxidative resistance of high-density polyethylene geomembranes. Polym. Degrad. Stabil..

[15] Ewais A.M.R., Rowe R.K., Rimal S., Sangam H.P. (2018). 17-year elevated temperature study of HDPE geomembrane longevity in air, water and leachate. Geosynth. Int..

[16] Rowe R.K., Rimal S., Sangam H. (2009). Ageing of HDPE geomembranes exposed to air, water and leachate at different temperatures. Geotext. Geomembr..

[17] Abdelaal F.B., Morsy M.S., Rowe R.K. (2019). Long-term performance of a HDPE geomembrane stabilized with HALS in chlorinated water. Geotext. Geomembr..

[18] Abdelaal F.B., Rowe R.K. (2019). Degradation of an HDPE geomembrane without HALS in chlorinated water. Geosynth. Int..

[19] Clinton M., Rowe R.K. (2024). Long-term durability of two HDPE geomembranes formulated with polyethylene of raised temperature resistance (PE-RT). Geotext. Geomembr..

[20] Samea A., Abdelaal F.B. (2023). Effect of elevated temperatures on the degradation behaviour of elastomeric bituminous geomembranes. Geotext. Geomembr..

[21] Plota A., Masek A. (2020). Lifetime Prediction Methods for Degradable Polymeric Materials—A Short Review. Materials.

[22] Cazzuffi D., Gioffrè D. (2020). Lifetime assessment of exposed PVC-P geomembranes installed on Italian dams. Geotext. Geomembr..

[23] Blanco M., Touze-Foltz N., Pérez Sánchez M., Redón-Santafé M., Sánchez Romero F.J., Torregrosa Soler J.B., Zapata Raboso F.A. (2018). Durability of reinforced PVC-P geomembranes installed in reservoirs in eastern Spain. Geosynth. Int..

[24] Koerner R.M., Lord A.E., Hsuan Y.H. (1992). Arrhenius modeling to predict geosynthetic degradation. Geotext. Geomembr..

[25] Hsuan Y.G., Koerner R.M. (1998). Antioxidant depletion lifetime in high density polyethylene geomembranes. J. Geotech. Geoenviron. Eng..

[26] Ito M., Nagai K. (2007). Analysis of degradation mechanism of plasticized PVC under artificial aging conditions. Polym. Degrad. Stabil..

[27] Ivanič A., Lubej S. (2022). Durability and degradation of pvc-p roofing membrane—Example of dynamic fatigue testing. Polymers..

[28] (2019). Standard Test Method for Measuring the Nominal Thickness of Geosynthetics.

[29] (2024). Standard Test Method for Measuring Mass Per Unit Area of Geotextiles.

[30] (2020). Standard Test Method for Determining Tensile Properties of Nonreinforced Polyethylene and Nonreinforced Flexible Polypropylene Geomembranes.

[31] Merry S.M., Bray J.D. (1996). Geomembrane response in the wide strip tension test. Geosynth. Int..

[32] Rowe R.K., Ewais A.M.R. (2014). Ageing of exposed geomembranes at locations with different climatological conditions. Can. Geotech. J..

[33] e Silva R.A., Abdelaal F.B., Rowe R.K. (2025). A 9-year study of the degradation of a HDPE geomembrane liner used in different high pH mining applications. Geotext. Geomembr..

[34] Blanco M., Castillo F., Touze-Foltz N., Amat B., Aguiar E. (2015). Behaviour of an EPDM geomembrane 18 years after its installation in a water reservoir. Int. J. Geomate..

